# Early phase of effective treatment induces distinct transcriptional changes in *Mycobacterium tuberculosis* expelled by pulmonary tuberculosis patients

**DOI:** 10.1038/s41598-021-96902-7

**Published:** 2021-09-08

**Authors:** Ambreen Shaikh, Kalpana Sriraman, Smriti Vaswani, Vikas Oswal, Sudha Rao, Nerges Mistry

**Affiliations:** 1 The Foundation for Medical Research, Dr. Kantilal J. Sheth Memorial Building, 84-A, RG Thadani Marg, Worli, Mumbai, Maharashtra 400018 India; 2 Vikas Nursing Home, Shivaji Nagar, Govandi, Mumbai, India; 3 Genotypic Technology Pvt Ltd, RMV Second Stage, Bangalore, India

**Keywords:** Bacterial genes, Pathogens

## Abstract

Effective treatment reduces a tuberculosis patient's ability to infect others even before they test negative in sputum or culture. Currently, the basis of reduced infectiousness of the *Mycobacterium tuberculosis* (Mtb) with effective treatment is unclear. We evaluated changes in aerosolized bacteria expelled by patients through a transcriptomic approach before and after treatment initiation (up to 14 days) by RNA sequencing. A distinct change in the overall transcriptional profile was seen post-treatment initiation compared to pretreatment, only when patients received effective treatment. This also led to the downregulation of genes associated with cellular activities, cell wall assembly, virulence factors indicating loss of pathogenicity, and a diminished ability to infect and survive in new host cells. Based on this, we identified genes whose expression levels changed with effective treatment. The observations of the study open up avenues for further evaluating the changes in bacterial gene expression during the early phase of treatment as biomarkers for monitoring response to tuberculosis treatment regimens and provide means of identifying better correlates of Mtb transmission.

## Introduction

Effective treatment is an important measure to halt the transmission of tuberculosis (TB)^1,2^. Over the years, several studies have tried to understand the transmission of TB from drug-susceptible (DS) or multidrug-resistant (MDR) patients on standard treatment regimens using human to guinea pig transmission models or contact tracing cohorts^3–6^. These studies reported that effective treatment could abolish transmission by patients with DS-TB even before sputum conversion was achieved. Even in MDR treatment, seminal studies have shown that effective treatment, as little as 24 h after the start of treatment, rendered MDR-TB patients non-infectious^3,4^. Dharmadhikari et al. (2014)^3^ proposed that the most plausible mechanism impacting the infectiousness of *Mycobacterium tuberculosis* (Mtb) bacilli without affecting its viability may be the rapid gene expression changes associated with treatment, which impact virulence. However, no molecular study to date has confirmed this theory, and the pathogen-specific processes that are perturbed when Mtb is treated with minimal effective or ineffective drugs remain unknown.

Antimycobacterial drugs can affect the Mtb gene expression dynamics, leading to altered cellular state or transcriptional adaptation and antibiotic tolerance^7^. Most studies have analyzed the transcriptional response of Mtb to these drugs and shown that drug-induced changes correspond to the drug's mode of action^7–9^. For instance, drugs like isoniazid and ethionamide which inhibit cell wall synthesis, modulates genes involved in the FASII pathway, lipid and intermediate metabolism, and transcription regulators^8–11^. Antimycobacterial drugs are also shown to modulate stress response genes: kanamycin and bedaquiline induce DosR^12,13^, linezolid induces chaperone encoding genes^14^ and quinolones induce an SOS response^15^. These drug-induced changes affect cell wall permeability, transport of membrane proteins, virulence, and antibiotic tolerance^7,8,16^. However, these studies have analyzed the effect of a single drug^9,14,17,18^ or a combination of drugs^8,11,19^, in vitro under standard growth conditions, which may not reflect the in vivo changes. Furthermore, Mtb transcriptional patterns in the clinical specimen are distinct from those observed in vitro or in animal models^10^. Few studies have investigated the transcriptional signature of Mtb from direct clinical samples that better represent the in vivo status during infection, transmission, and treatment^10,20–22^. In sputum Mtb, drug treatment was shown to upregulate transcription and sigma factors, efflux pumps, and stress response while downregulating genes involved in growth and metabolism^22^. The focus of these studies was on the long-term effect of TB treatment on bacterial survival or the adaptive ability of Mtb to escape the effects of antimycobacterial compounds (persisters)^20,22,23^. Thus, there is a dearth in understanding of the transcriptional response that impacts the infectiousness of Mtb, especially during the early phase of treatment.

The present study investigated the effect of early treatment (the first 14 days of treatment) on Mtb from pulmonary TB patients undergoing first-line anti-TB drug therapy. Traditionally, Mtb transcriptional studies have utilized sputum from patients as a source for the bacilli, although sputum Mtb can only be a surrogate marker for transmission^24^. As respiratory aerosols with Mtb are the route for infectiousness in tuberculosis; it is essential to examine Mtb expelled by patients through respiratory particles to gain insights into the bacterial transcriptome potentially involved in transmission. Our previous study had demonstrated that N95 masks lined with capture membrane were effective in entrapping respiratory particles containing infectious aerosols and study Mtb transcripts quantitatively^25^. The current study utilized this approach and profiled the transcriptome of the Mtb expelled by DS and MDR patients during the first 14 days of first-line anti-TB drug therapy through RNA sequencing (Investigation set). We hypothesized that the bacterial gene expression profile would be different when the Mtb encountered "effective" vs. "ineffective" or inadequate treatment. Therefore, we carefully classified the respiratory samples into effective and ineffective treatment sets considering the treatment received, genotyping using whole-genome sequencing (WGS), following up on treatment adherence and completion, and the final pronouncement of treatment outcomes by the treating physician. The transcriptional changes in aerosolized Mtb observed in response to treatment were also validated in separate patient sets (validation set) using quantitative reverse transcriptase-polymerase chain reaction (qRT-PCR).

## Results

### Mtb transcriptional signature in response to treatment by RNA sequencing

The investigation set consisted of mask samples from nine treatment-naïve, confirmed pulmonary TB patients who received first-line anti-TB drug therapy (Isoniazid, Rifampicin, Pyrazinamide, and Ethambutol, HRZE). Supplementary Table S1 provides a detailed description of patient profiles. The patients in the effective treatment group (GeneXpert DS + HRZE treatment, n = 7, median age 28 years) had mild to moderate disease (chest X-ray impression as noted by the physician), median X-ray score of 7.5 (4–11), BMI of 19 (18–21) and had no known comorbidities. The patients with ineffective treatment (GeneXpert DR + HRZE treatment, n = 2, age 19 and 21 years) similarly had mild to moderate disease, X-ray score of 10 and 6, BMI of 18 and 24, and one of the patients had diabetes. In addition, all patients showed culture positivity up to 14 days of treatment, indicating that patients in both effective and ineffective treatment groups continued to carry viable bacteria for this timeframe (Supplementary Fig. S1). Strain typing using WGS showed that the MTB isolated from the patient samples with effective treatment belonged to Beijing (n = 1), Delhi-CAS (n = 2), or European American strains (n = 3) while Mtb isolated from patients with ineffective treatment belonged to Beijing and Delhi-CAS lineage. During the study period, the field staff noted on adherence to treatment through regular follow-up phone calls. At the time of writing this manuscript, all patients in the effective treatment group had completed their six-months anti-TB drug therapy and reported no relapse. Also, the patients in the ineffective treatment group were shifted from first-line treatment to MDR treatment regimen after DST confirmation and were in the process of completing their prescribed regimen.

To investigate the global gene expression changes associated with the early phase of treatment, we compared the mRNA transcriptional signature of Mtb on 1, 3, 5, 7, and 14 days of treatment with Mtb transcription at pretreatment conditions. Using Illumina sequencing, a median of 3.816E+07 (2.863E+07–4.483E+07) high-quality processed reads were generated from each sample (Supplementary Table S2) and aligned to the Mtb H37Rv genome — 590,217 (65,229–4,731,000) reads aligned to the reference Mtb in the pretreatment samples whereas 319,747 (128,230–781,418) reads aligned in the post-treatment initiation samples. HTSeq gene count analysis showed an almost five-fold reduction in the number of genes covered after patients started the anti-TB therapy than pretreatment (median 1988 (130–3925) genes pretreatment v/s 340 (203–1285) genes post-treatment initiation) (Supplementary Table S2). One patient set showed very low gene coverage even at pretreatment (≤ 100 genes) and was not considered for further analysis.

### Effective treatment brings distinct changes in the global gene expression profile of Mtb

**Figure 1:**
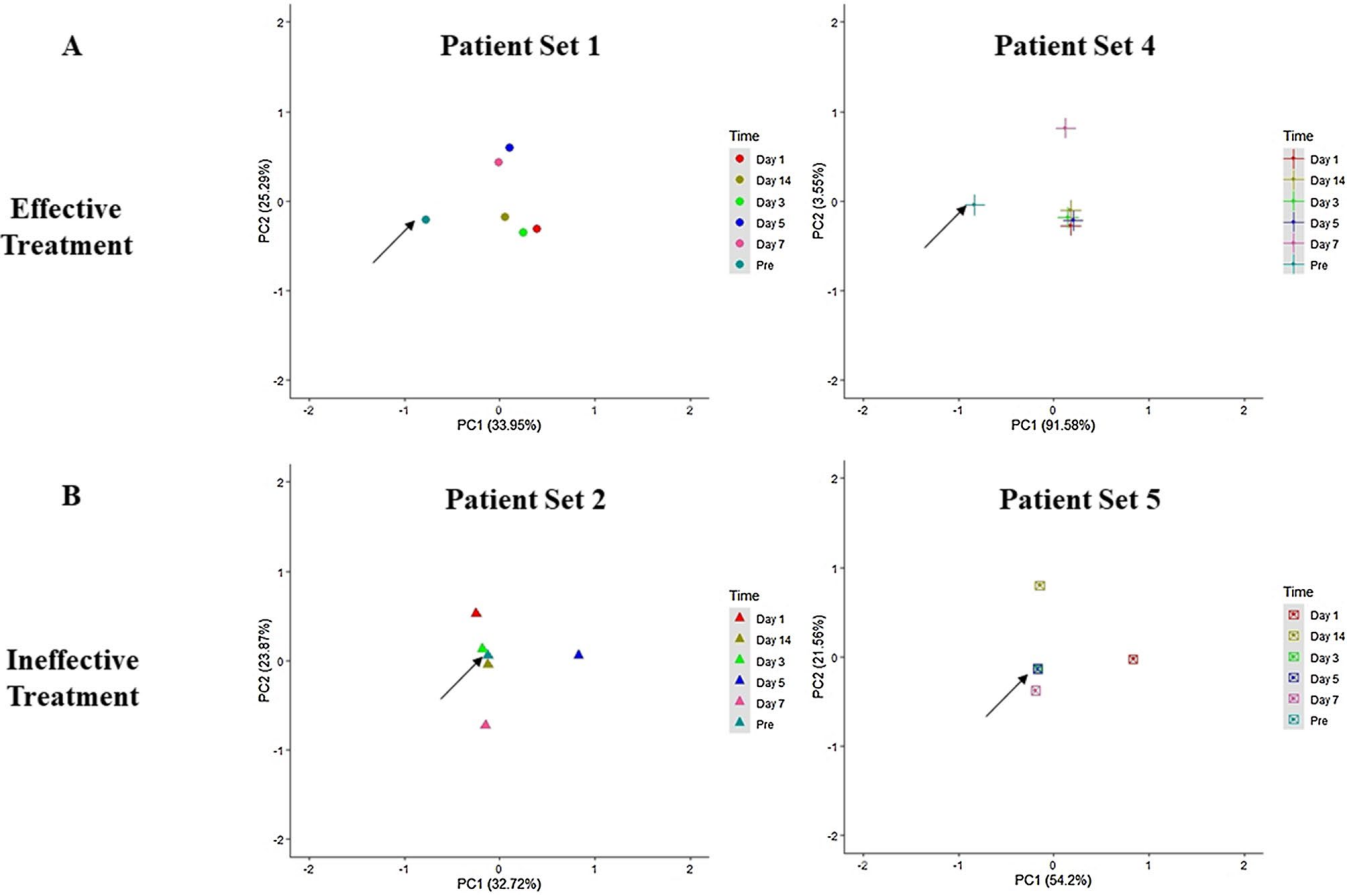
Principle Component Analysis (PCA) plots. The plots show the first and second principal components of the Mtb transcriptional profile from each patient at different time points of treatment (Pretreatment to 14 days’ post-treatment initiation). PCA shows the complete transcriptome in a single point, and each point on the plot represents the Mtb mRNA profile derived from the one patient mask sample at each time point. The top row (**A**) represents PCA plots of patient sets with effective treatment, and the bottom row (**B**) represents PCA plots of patient sets with ineffective treatment. The arrow marks the pretreatment point on the PCA plots.

We performed unsupervised principal component analysis (PCA, Fig. 1) and plotted the first and second principal components of Mtb transcriptional profile from each patient set — pretreatment and during the first two weeks of treatment. The representative PCA plots of individual patient sets in the effective treatment group showed that Mtb transcriptional profile post-treatment initiation clustered separately and was distinct from the transcriptional signature pretreatment (Fig. 1A). Interestingly, in the ineffective treatment group, the pretreatment transcriptional signature clustered along with the transcriptional signature on days 3–14 post-treatment initiation (Fig. 1B). In this group, the transcriptional signature on day one of treatment did not cluster with the pretreatment profile in any of the patient sets (Fig. 1B). Therefore, the clustering of points in the PCA plots suggests that Mtb's response to the early phase of drug therapy differs between patients receiving effective and ineffective treatment. Supplementary Fig. S2 shows the PCA plot of the pooled samples patient set.

### Effective treatment perturbs the expression of distinct functional categories reflecting reduced infectiousness potential

The PCA plots showed an alteration in the overall transcriptomic profile only in response to effective treatment. To understand the impact of treatment on the function of the bacilli, differential gene expression analysis was performed at each time point post-treatment initiation compared to its respective pretreatment levels. Differentially expressed genes were filtered out using a false discovery rate (FDR) of < 0.05 and log2 fold change > 1.5 or < 1.5 for biological significance. With effective treatment, 157–1439 genes were dysregulated after initiation of treatment, while with ineffective treatment, only 28–149 were dysregulated (Supplementary Table S3). The functional significance of the differentially expressed genes was investigated in the ways described below.

1. Functional categories as classified by Mycobrowser (https://mycobrowser.epfl.ch)
2. Gene ontology (biological process) and pathway (KEGG^26^) specific enrichment using DAVID web resource^27^
3. Functional gene sets involved in growth, infection of host cells, and stress response

Functional category analysis showed that the most dysregulated genes belonged to the categories of the cell wall and cell wall process, conserved hypotheticals, and intermediary metabolism and respiration (Table 1). This was expected due to the large number of genes that were catalogued in these categories. Further considering the category size, we noted that with effective treatment, the functional categories of lipid metabolism, PE/PPE, and information pathway were significantly dysregulated after treatment (Table 1, Supplementary Table S4). Comparatively, with ineffective treatment, a significant alteration was seen only in the categories of conserved hypotheticals, lipid metabolism, and information pathways during 1 to 3 days of treatment (Table 1, Supplementary Table S4).

**Table 1:** Distribution of genes based on functional categories as categorized by mycobrowser.

Functional categories	Rx-1D				Rx-3D				Rx-5D				Rx-7D				Rx-14D			
	Effective treatment		Ineffective treatment		Effective treatment		Ineffective treatment		Effective treatment		Ineffective treatment		Effective treatment		Ineffective treatment		Effective treatment		Ineffective treatment	
	N	%	N	%	N	%	N	%	N	%	N	%	N	%	N	%	N	%	N	%
Cell wall and cell wall process	40	**6**	20	**3**	*125*	**18**	*14*	**2**	*250*	**36**	5	**1**	52	**8**	4	**1**	22	**3**	4	**1**
Conserved hypotheticals	76	**7**	*26*	**3**	*126*	**12**	*20*	**2**	*400*	**39**	5	**0**	*48*	**5**	4	**0**	*30*	**3**	2	**0**
Information pathways	*15*	**7**	14	**6**	*41*	**18**	*15*	**7**	*78*	**34**	4	**2**	18	**8**	0	**0**	11	**5**	5	**2**
Insertion sequences and phages	*16*	**22**	2	**3**	7	**9**	1	**1**	*27*	**36**	0	**0**	5	**7**	0	**0**	1	**1**	0	**0**
Intermediary metabolism and respiration	*39*	**5**	35	**4**	118	**14**	28	**3**	*273*	**33**	3	**0**	57	**7**	11	**1**	*30*	**4**	6	**1**
Lipid metabolism	10	**4**	*19*	**8**	*48*	**21**	14	**6**	30	**13**	7	**3**	*25*	**11**	2	**1**	*18*	**8**	2	**1**
Not classified	*48*	**12**	14	**4**	*51*	**13**	11	**3**	135	**34**	6	**2**	*21*	**5**	4	**1**	*24*	**6**	6	**2**
PE/PPE	12	**8**	10	**6**	21	**13**	11	**7**	*72*	**46**	0	**0**	16	**10**	3	**2**	8	**5**	1	**1**
Regulatory proteins	14	**8**	7	**4**	*30*	**17**	2	**1**	74	**42**	0	**0**	12	**7**	0	**0**	4	**2**	1	**1**
Virulence detoxification adaptation	*14*	14	4	4	22	22	4	4	38	38	0	0	12	12	0	0	*8*	**8**	1	**1**

N -Detected genes in each category, % percentage; numbers in bold represent the percentage of dysregulated gene calculated based on the number of genes in the particular functional category, numbers in italics indicate the significantly dysregulated functional categories. Rx-1d–Rx-14d – Days post-treatment initiation.

The bidirectionally regulated genes were further investigated to check for gene ontology (biological process) and pathway (KEGG^26^) specific enrichment using the DAVID web resource^27^. Table 2 lists only the significantly enriched (FDR < 0.05) biological process/pathways, and Supplementary Table S5 shows the complete enrichment profile. This analysis showed that with effective treatment, the down-regulated genes were significantly enriched in the biological process of growth, response to hypoxia, and pathogenesis on days 1, 3, 5, and 7 of treatment and in the ESX/Type VII secretion system on days 3 and 14 of treatment. The down-regulated genes were enriched in ribosome pathways, while upregulated genes were enriched in ABC transporters on day 5 of treatment. In the ineffective treatment group, we observed no significant enrichment in biological processes on any of the days' post-treatment initiation.

**Table 2:** Enrichment analysis for biological process and KEGG pathways.

Days post Rx initiation	GO term	Count	*p*-value	Fold enrichment	FDR
**Effective treatment: downregulation**					
Rx-1D	Growth	34	5.60E–04	1.7	3.40E–02
	Response to hypoxia	11	2.90E–06	6.6	3.50E–04
	Pathogenesis	13	1.10E–03	2.9	4.40E–02
Rx-3D	Growth	93	7.60E–09	1.7	1.80E–06
	Pathogenesis	34	4.40E–08	2.7	5.20E–06
	Protein secretion by the type VII secretion system	7	1.00E–04	7.4	7.10E–03
	Response to heat	8	1.20E–04	6	7.10E–03
Rx-5D	Translation	32	2.20E–06	2.3	6.40E–04
	Growth	161	3.10E–06	1.3	6.40E–04
	Response to hypoxia	25	6.90E–06	2.5	9.60E–04
Rx-7D	Growth	44	6.50E–06	1.8	1.00E–03
	Response to hypoxia	9	6.00E–04	4.5	4.80E–02
Rx-14D	Protein secretion by the type VII secretion system	5	6.30E–05	19.9	5.70E–03
**KEGG pathway**					
Rx-5D	Ribosome	33	5.20E–09	2.7	4.80E–07
**Effective treatment: upregulation**					
*KEGG pathway*					
Rx-5D	ABC transporters	24	4.40E–05	2.4	4.00E–03
**Ineffective treatment: upregulation**					
*KEGG pathway*					
Rx-7D	Biosynthesis of antibiotics	5	2.40E–03	4.50	4.20E–02

Enrichment of genes in biological process and KEGG^26^ pathway was performed using the functional annotation tool of the Database for Annotation, Visualization, and Integrated Discovery (DAVID) web interface v 6.8^27^. Rx- Treatment, Rx-1D to Rx-14D – days post-treatment initiation, *p*-Value < 0.05 considered significant, *FDR* false discovery rate.

**Figure 2:**
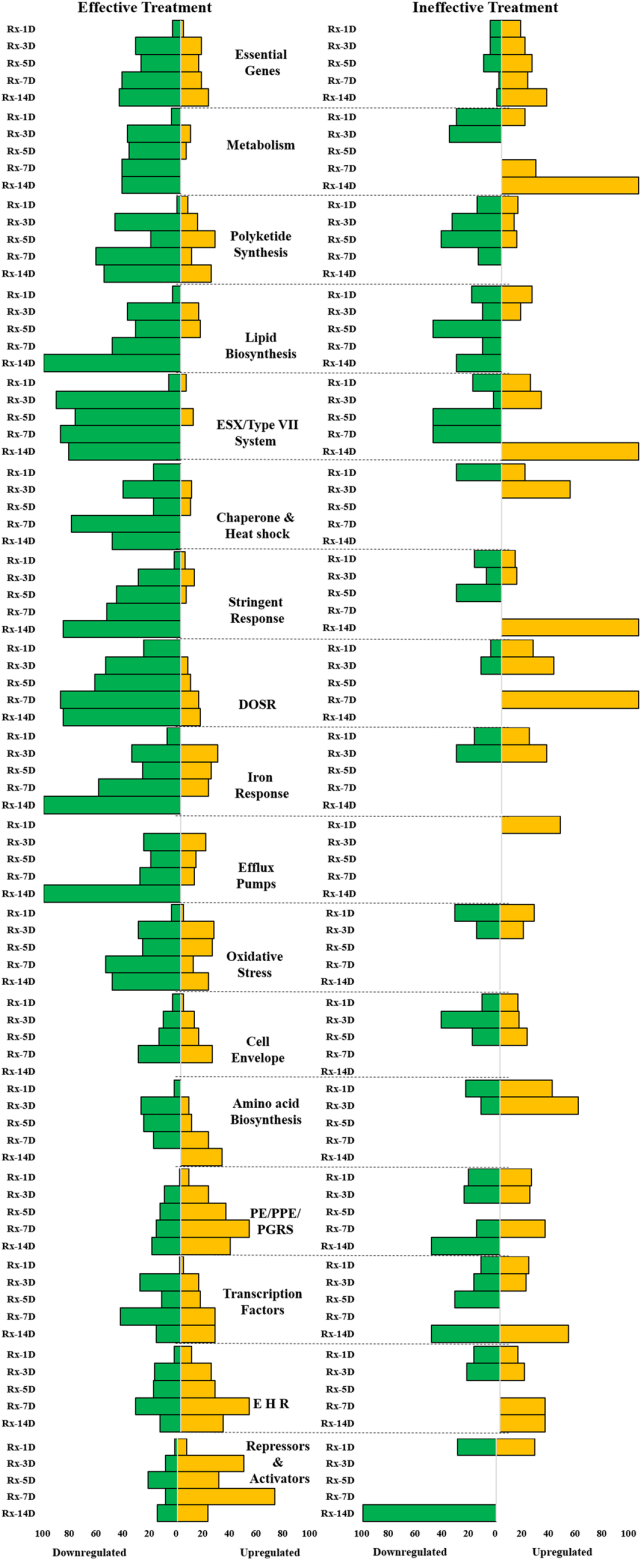
Change in expression of selected functional categories of genes after initiation of anti-tuberculosis treatment. The graphs illustrate the percentage of genes in each functional category significantly up- (yellow) or down- (green) regulated each day relative to pretreatment expression. The left and right side graphs represent the alterations observed in gene expression with effective and ineffective treatment, respectively. The dashed line demarcates each of the functional categories. The percentage of genes was calculated by dividing the number of genes up or down-regulated by the number of genes detected. This calculation was for genes detected at each profiled time point in the functional group.

**Figure 3:**
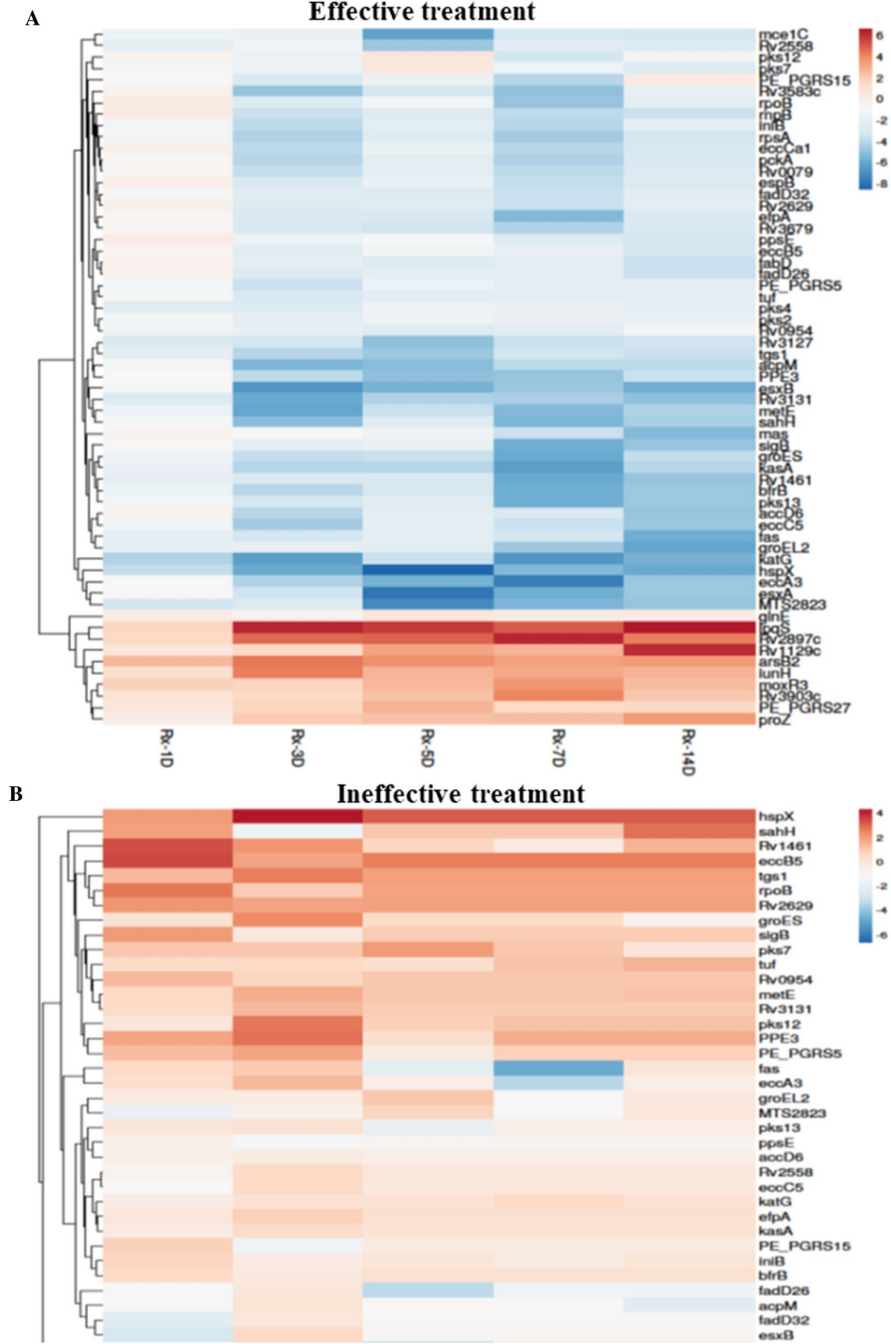
Heat map of fold-change genes differentially expressed between Days 1 to 14 of treatment. The top image (**A**) denotes the heat map of Mtb-specific genes evaluated in patients who received effective treatment, and the bottom image (**B**) denotes the heat map of Mtb-specific genes evaluated in patients who received ineffective treatment. The heat maps were generated with log2 fold change values using the Clustvis web tool. No scaling was applied to rows. Imputation was used for missing value estimation. Rows were clustered using Euclidean distance and average linkage.

During successful infection, Mtb actively transcribes genes involved in its fortification in the host cell, its survival under a stressful microenvironment, and evading the host immune system^21^. To understand the impact of treatment on infectiousness, survival, and stress response, we investigated the gene expression profile changes of specific functional gene categories^28^. We analyzed the day-wise expression of genes in 28 different functional gene sets (Supplementary Table S6) and observed significant alteration in 15 of these gene sets (Fig. 2). Additionally, we mapped a few key repressed and induced genes from these sets in both treatment groups. Figure 2 shows the difference in the expression profile in the 17 gene sets in the two treatment groups and Fig. 3 shows the expression of individual genes in the two treatment groups.

#### Growth and cell wall synthesis

With effective treatment, the expression of the majority of essential genes^29^ that are required for the Mtb growth and genes associated with the metabolic process (TCA cycle, Glyoxylate bypass cycle, ATP synthesis, and aerobic respiration) were downregulated immediately after the start of treatment (day 1) and continued to remain suppressed even up to day 14 of treatment (Figs. 2 and 3A). Another predominant downregulation was seen in genes involved in polyketide synthesis (*pks2,4,12,13*), lipid biosynthesis (*fas, accD6, fabD, acpM, kas A, fadD26, fadD32*), and efflux pumps (*efpA*, *iniB*, Figs. 2 and 3A), suggesting dysregulation in cell wall synthesis. In contrast, in the ineffective treatment group, we observed an initial suppression of genes mentioned in the above categories; however, between 7–14 days of treatment, some essential genes and metabolic processes were induced (Figs. 2 and 3B). Interestingly, in the ineffective treatment group, the expression of efflux pump genes was induced immediately after treatment (Fig. 2). Apart from the aforementioned functional categories, aspartate and glutamate synthesis, sulphur metabolism, and gluconeogenesis were also down-regulated with effective treatment (Fig. 3A).

#### Virulence and stress response

The reduction in cellular activity was accompanied by the downregulation of genes involved in regulating virulence and infectiousness mechanisms. For instance, we observed that genes related to the highly immunogenic ESX system and a few PE/PPE were downregulated upon effective treatment initiation. Also, the iron assimilation and iron cluster genes (*bfrB, Rv1461, Rv1462*) and chaperone and heat shock proteins (*groEL2, groES, hspX*) that are required for survival in the host cell were suppressed through day 14 (Figs. 2 and 3A). Moreover, *mts2823*, a small non-coding RNA and a known regulator, was immediately suppressed on treatment and remained suppressed through day 14 (Fig. 3A). Various studies have shown that stress response (like that caused by anti-TB drugs or host) induces or represses the expression of a specific set of genes^30–32^. In the current study, the stringent and oxidative response genes^30,32^ were significantly downregulated while enduring hypoxic response (E H R^31^) genes, anion transport gene *arsB2,* and few PE/PPE genes (*PE/PGRS 15, 27*) were upregulated (Figs. 2 and 3A). In addition, we observed that the dosR regulon, which is generally induced by the stress response, was suppressed throughout the mapped treatment days (Figs. 2 and 3A). The response regulators had a mix of up-and-down-regulated genes; genes like *moxr3*, *Rv1129c* (Fig. 3A) were upregulated, while genes like *Rv3583c, whiB3* (not shown in the heat map) were down-regulated.

Cumulatively these results suggest that though some Mtb survive initial chemotherapy as indicated by culture positivity up to 14 days of treatment, effective treatment significantly represses key functional categories, processes, and pathways that may severely diminish their pathogenesis and ability to infect a new host.

### Investigating drug-induced changes and persister phenotype in aerosolized Mtb

#### Drug-induced changes

Exposure of Mtb in vitro to rifampicin, isoniazid, and ethambutol is known to modulate the activity of specific gene clusters^8^. Here, we mapped the expression pattern of these specific gene clusters in Mtb derived from patient aerosols at 1, 3, 5, 7, and 14 days after the start of effective treatment. In our Mtb transcriptome post-effective treatment initiation, we detected transcripts involved in the activity of rifampicin, isoniazid, and ethambutol (Supplementary Table S10). The heat map of the transcriptional profile showed that 50% of these transcripts showed a sustained down-regulation between days 3–14 of treatment (Fig. S3). The down-regulated Mtb transcripts were a part of—(A) heat shock proteins and ribosome synthesis associated with rifampicin treatment (B) FASII pathway, fatty acid synthesis and modification, and polyketide synthesis associated isoniazid and ethambutol treatment. The results indicate that specific drug-induced transcriptional changes are seen from day three and sustained even at the end of two weeks of treatment.

#### Evaluating the persister phenotype

Transcriptome profiling studies in sputum indicate that Mtb survive after two weeks of anti-TB treatment show drug-tolerant/persister phenotype on day 14^20,23^. Thus, we compared aerosolized Mtb transcriptomic profile with sputum Mtb or sputum derived Mtb described in these studies. The significantly up and down-regulated genes on day 14 in the aerosolized Mtb with effective treatment were compared with the following (i) Day 14 transcriptomic profile of sputa derived Mtb exposed to Rifampicin, Isoniazid, Streptomycin, and Ciprofloxacin in vitro^23^ (ii) Day 14 transcriptomic profile of sputum Mtb in patients on standard anti TB treatment (in vivo)^20^.

A four-dimensional Venn diagram (Fig. S4) summarizes the differences and similarities of the transcriptomic profiles. Only 7 out of the 69 repressed genes on day 14 in aerosolized Mtb overlapped with genes from the transcriptomic profile of sputa derived Mtb exposed to standard treatment in vivo. These genes were involved in the ESX system, intermediary metabolism, PE/PPE, and regulatory proteins, which are likely associated with Mtb pathogenicity. No overlap of genes was seen between the aerosolized Mtb and the in vitro transcriptional response. Figure S5 follows the expression pattern of these seven genes between days 1 to 14 post-treatment initiation in our data set. We observed that the down-regulation for most of these genes started on day 3 of treatment and was evident even on day 14.

### Validation of Key differentially regulated genes by qRT-PCR

Next, we investigated the key dysregulated genes that were affected by effective treatment but not by ineffective treatment and represent reduced infectiousness. We identified 16 genes (Table 3) belonging to the functional categories of virulence, host infection, and survival. These genes showed a distinct expression profile in response to effective treatment. We validated the expression pattern of these 16 genes in 10 independent patient sample sets (pretreatment and post-treatment initiation). The profile of patients in the validation set was similar to the investigation set. The patients (n = 10, median age 21 years) had mild to moderate chest X-ray impression, median X-ray score 9 (7–9), median BMI of 19 (17–21), no known comorbidities, and the Mtb strains were Delhi-CAS (n = 6) or European American (n = 2) or East African Indian Ocean (n = 1) (Supplementary Table S1).

**Table 3:** Genes selected for validation.

Expression pattern	Genes
Down regulated	*eccC5, esxB, pks13, fadD26, groEL2, mts2823, glnA1, katG, kasA, bfrB ,nrdF2, espB*
Up regulated	*moxR3, arsB2,glnE, ftsW*

**Figure 4:**
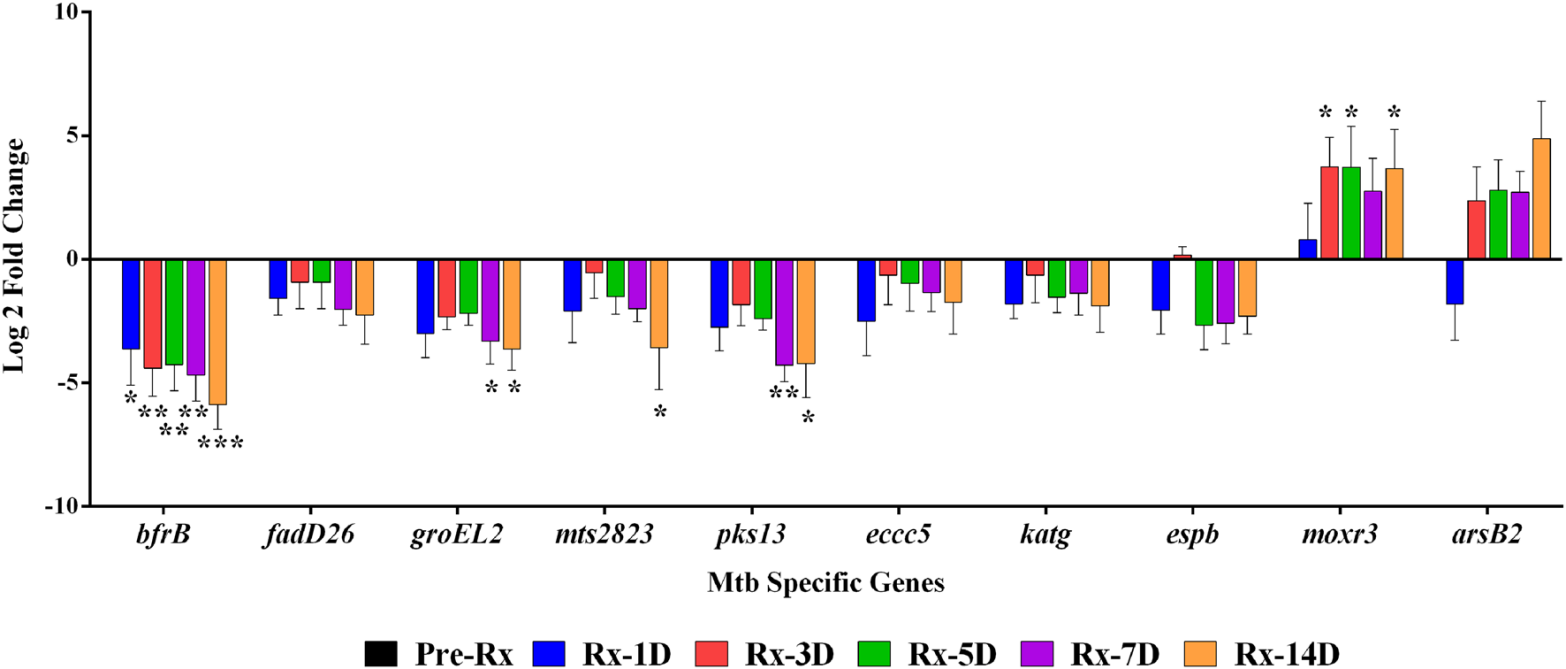
Validation of the ten differentially expressed genes in ten patient sets by qRT-PCR. The graph shows the change in Mtb-specific genes' expression during different treatment days in 10 patient samples relative to pretreatment. The data were normalized using *16S* as housekeeping, and relative gene expression was calculated by the ΔΔCt with pretreatment (Pre-Rx) as control. Asterisks indicate a significant difference in gene expression between days of treatment compared to pretreatment, with *p* < 0.05 considered statically significant. Average log2 fold change values are plotted; the error bars mark standard deviation. *p < 0.05, **p < 0.01, ***p < 0.001.

Of the 16 genes, the expression (pattern) of 10 genes could be validated (Fig. 4). These ten genes were detected in all pretreatment samples by qRT-PCR; however, with treatment initiation, we observed that the expression of a few genes could not be captured on days 7 and 14 of treatment. The genes *bfrB, mts2823, groeL2, eccC5, katG* were downregulated in all samples at all time-points. The genes *moxR3, espB, and pks13* were detected in 8/10 samples, while *arsB2 and fadD26* were detected only in 7/10 samples. Though the fold change expression levels varied, the upregulation or downregulation expression pattern of the validated genes from qRT-PCR was the same as RNAseq (Fig. 4). The *ftsW, glnA1, glnE, kasA, nrdF2*, and *esxB* showed expression in less than five sample sets and could not be validated.

## Discussion

Antibiotics can affect bacteria at many levels, and transcriptional profiling of Mtb in the presence of these drugs can shed light on their direct and indirect effects. Through RNA sequencing, we confirmed that the early phase of the first-line anti-TB drug therapy alters the global transcriptional signature of the Mtb that survives in patients after treatment initiation. This alteration in the transcriptome was seen as early as day one of treatment. Interestingly, we found Mtb remained transcriptionally active after exposure to two weeks of treatment but with widespread repression in key survival and virulence factors. The study conducted by Walter et al.^22^ bears highlighting here as one of the initial direct sampling studies that showed alteration in Mtb gene global expression profile in response to the early phase of treatment. The difference between the study by Walter et al. and ours is mainly the source of Mtb, the transcriptional profiling technique (RNAseq), and the experimental model of two treatment groups. The Walter et al., 2016 study used qRT-PCR and mapped the expression of Mtb-specific genes in bacilli isolated from human sputa, focusing on characterizing the persister Mtb population^22^. In the present study, we used respiratory aerosols exhaled or expelled by PTB patients as the sample source to understand the relevance of Mtb transcription to transmission. Additionally, our analysis was based on patients' treatment regimen—effective vs. ineffective regimen. Using this design, we found that changes in transcriptome profile with treatment (Fig. 1) and expression patterns of genes in various functional categories (Fig. 2) varied between treatment groups. This is the first study, to the best of our knowledge﻿ to show the changes in aerosolized Mtb transcriptome in response to the early phase of the first-line anti-TB treatment regimen in two different treatment groups.

The principal component analysis patterns give a bird's eye view of changes in transcriptional response^20^. The PCA plots of the patient sets in the effective treatment group show that even one day of treatment alters Mtb transcriptional response, and these changes are sustained till the end of two weeks of treatment. In contrast, the PCA plots of patient sets in the ineffective treatment group show altered Mtb response to drug therapy only at the 24-h time point and, the transcriptional signature in the latter days of treatment (3–14) was similar to pretreatment, suggesting no effect of drug therapy on the Mtb. A second seminal study by Honeyborne et al.^20^ mapped mRNA signatures of Mtb from sputa and showed that PC trajectories varied with patients and that the changing Mtb transcriptional profiles 0–2 weeks into chemotherapy could predict the efficacy of treatment six weeks later. Similarly, even in our study, the PCA patterns followed different paths in the effective and ineffective treatment groups (Fig. 1). This suggests that Mtb's response to drug therapy may differ between the two groups and that these changes in the bacterial mRNA signature may be of prognostic value as an early predictor of treatment efficacy.

The differentially expressed genes in the two groups may provide clues on the adaptation of Mtb in response to different treatment types. To understand the impact of the anti-TB treatment on the infectiousness and survival of Mtb, we evaluated possible mechanisms that arose due to the altered Mtb transcriptional signature. The transcriptomic profile of Mtb post-treatment initiation showed downregulation of *bfrB, groEL2, fadD26, pks13, and eccC5*. The gene *bfrB* encodes the major iron storage protein of Mtb. It functions as an Mtb host interaction effector molecule that subverts the host immune response and promotes Mtb survival by inhibiting cytokine production^33^. With the downregulation of *bfrB* post-treatment initiation, the host immune response may not be subverted, affecting Mtb survival and aiding in the clearance of Mtb. Another validated gene, *groeL2,* encodes chaperone proteins, which are potential virulence determinants and facilitate the blocking of macrophage apoptosis^34^. The *groeL2* gene is reported to be upregulated during TB infection and downregulated in response to rifampicin^8,21^. In this study, we hypothesize that the immediate and sustained down-regulation of *groEL2* with treatment corresponds to lower levels of chaperone proteins, leading to lesser blocking of macrophage apoptosis and increased clearance of Mtb. The downregulation of *groeL2* also signifies reduced virulence and infection. The genes *fadD26* and *pks13* are involved in the biosynthesis of PDIM^28,35^, which plays a role in cell wall modelling and virulence^35^. Mtb post-initiation of effective treatment showed downregulation of these genes, suggesting increased cell barrier permeability which could help anti TB drugs gain access to bacterial targets and consequently reduce the Mtb's infective potential. Furthermore, the repression of *eccC5* genes affects the *eccB5-eccC5* operon, a part of the ESX-5 system, which is detrimental to the transport of PE/PPE proteins and eventually affects the Mtb's ability to infect new host cells^36^.

At least one-third of the Mtb transcriptome, including rRNA, belongs to non-coding RNAs^37^. Studies have shown that the Mtb transcriptome has a considerable expression of non-coding transcripts under different in vitro stress conditions, suggesting their role in mycobacterial survival^37^. In this study, five transcripts encoding small ncRNA were detected (*mts0858, mts1082, mts1338, mts2823, and mts2975*). Only mts2823 from the detected ncRNAs showed a consistent, significant downregulated profile during the early phase of effective treatment. In contrast, with ineffective treatment, suppression of *mts2823* was short-lived, and by day five, its expression was upregulated, indicating that in the absence of proper treatment, Mtb adapts to ensure its survival. Overexpression of *mts2823* upregulates two genes and downregulates about 300 genes, including methyl citrate synthase^38^. The instability in the expression of *mts2823* via its downstream targets could lead to deregulation of RNA polymerases complex, affecting the bacilli's ability to respond to its current growing conditions^38^. In this study, the downregulation of *mts2823* in response to treatment may also have a similar effect; however, additional studies are required to understand whether this ncRNA is a regulator for other altered Mtb transcripts observed in our studies.

Boschoff et al. have mapped the specific transcriptional response of Mtb to drugs, drug combinations, and growth conditions^8^. The rifampicin-specific response showed an overall global downregulation of ribonucleotide reductase, heat shock proteins, and ribosomal proteins^8^. The transcriptional response of Isoniazid and Ethambutol had a characteristic induction of genes of *kas* operon involved in the FASII pathway, *efpA*, and *fbpC*^8^. In the transcriptional profile of our aerosolized Mtb post-treatment, the rifampicin-specific repressive expression profile was similar. However, the transcriptional response to isoniazid and ethambutol described by Boschoff et al., 2004 was not seen in aerosolized Mtb. The repression of isoniazid-specific genes in the aerosolized Mtb suggests that transcription patterns seen in vitro may not be seen in vivo and supports previously published studies that have made similar conclusions^10^.

We also observed certain similarities and differences in comparing the transcriptional profile of aerosolized Mtb with sputum Mtb. Overall, compared to untreated Mtb, the transcriptional signature of both aerosolized and sputum Mtb showed global downregulation of genes. The repression of metabolism-specific genes, lipid biosynthesis, and genes that regulate growth and transcription were seen in both the transcription profiles. One of the features of sputum Mtb post-treatment was the presence of a drug-tolerant Mtb population characterized by the induction of efflux pumps, dosR regulon, and sigma factors^20,22,23^. This specific response was not observed in the transcriptional profile of aerosolized Mtb post-effective treatment. Induction of dosR is necessary for Mtb persistence as it modulates the Th1 response to ensure the survival of the bacilli^39^. In the absence of induction of dosR, as seen in aerosolized Mtb, Th1 response is activated, affecting the formation of persister phenotype. In addition, the minimal overlap of genes between our transcriptome and published persister transcriptome^20,23^, further suggests the absence of Mtb with drug-tolerant phenotype in aerosols exhaled by patients receiving effective treatment. Moreover, induction of dosR is not seen when the oxygen tension is high. In case of TB infection, these conditions are only met in caseous lesions open to airways during aerosol transmission^31^. From the absence of induction of dosR in our transcriptome profile, we can infer that the aerosol capture model provided the closest approximation of Mtb involved in aerosol transmission.

Based on these mechanisms, we can hypothesize that effective treatment leads to the downregulation of factors required for Mtb survival in the host and the infectiousness/virulence machinery required for infecting a new host. In contrast, ineffective treatment does not overtly perturb the transcriptome, ensuring the survival of Mtb and may even contribute to the persister population's development. This study's results directly support the observations made by human to animal transmission studies^3,4,6^, where transmission of TB from patients occurred only when patients received ineffective treatment and patients who received effective treatment could not transmit despite expelling viable bacteria. Dharmadhikari et al.^3^ proposed that transcriptional alteration in Mtb in response to treatment may be responsible for abolishing the bacilli's transmission capability. Our study's data support this hypothesis and show that treatment alters the transcriptional signature of aerosolized Mtb, affecting its infectiousness potential. However, this needs to be further validated. The genes identified to be affected by effective treatment may be modified using gene-editing technologies and conduct challenge experiments in animal models to study its relevance to transmission and infection directly.

The study is limited by the sample size of the investigation set, especially for the ineffective treatment group (n = 2). Though we have tried to validate the most important genes in a more extensive validation set of ten patients, we could not include MDR patients receiving ineffective treatment in this set. It was difficult to capture such patients as the standard practice in clinical settings now includes upfront GeneXpert and the correct treatment initiation forthwith. Due to the small MDR patient set (n = 2), the study's outlook is more from the perspective of DS patients receiving effective treatment and not a direct comparison between DS and DR patients. Also, we observed a lesser number of transcripts in MDR patient samples. One possible explanation could be the difference in the number of viable Mtb exhaled by DS and MDR patients. To this effect, a recent study by Theron et al., 2020, has shown that MDR patients have a lower number of culture-positive Mtb bacilli in their cough aerosols^40^. Subsequent studies with a larger sample size of MDR patients with unsuspected XDR could contribute to a more substantial mechanistic difference in mRNA signatures of aerosolized Mtb between the patient groups. Moreover, the Mtb was studied in a culture-free manner directly from the PTB patients' respiratory aerosols, leading to low input RNA and comparatively less coverage of genes relative to cultured isolates, but possibly leaning to a more representative picture of in situ conditions.

In this study, we have described various possible mechanistic alterations to Mtb machinery that occur on receiving effective treatment. The next steps would be validating these mechanisms in vitro or in animal challenge models and investigating the transcriptional signatures when Mtb with an altered transcriptome infects a new host. In summary, using RNAseq, we provided a comprehensive view of the transcriptome landscape of aerosolized Mtb in the presence of effective and ineffective treatment. The bacterial transcriptional profile suggested that effective treatment represses the infectious potential of Mtb. Also, Mtb modulates different responses to effective and ineffective treatment. This opens avenues for conducting additional studies to validate potential gene candidates with differential expression patterns in treatment groups as biomarkers for treatment efficacy. In terms of clinical implications, we advocate that mapping the early alterations in Mtb transcriptome within two weeks of treatment and its associated transcriptional signature may be the way forward to gauge the new or additional treatment regimens' effectiveness more quickly and reliably during clinical trials of such regimes. These insights into yet unexplored niche of aerosolized Mtb transcriptome will help elucidate mechanisms behind the transmission of the disease.

## Materials and methods

### Patient recruitment and study design

The study was undertaken after approval by the Human Ethics Committee at the Foundation of Medical Research, Mumbai (FMR/IEC/TB/01/2017**)**. All methods were performed in accordance with the relevant guidelines and regulations. Informed consent was obtained from all participants before enrolling them in the study. From March 2018 through February 2020, we recruited, through sequential sampling, fifty presumptive, previously untreated pulmonary TB patients visiting the private TB clinics at Dharavi and Govandi in Mumbai. Mask (for respiratory aerosols) and sputum samples were collected at six different time points from each patient, i.e., before initiation of treatment (herein referred to as pretreatment) and on completion of 1,3,5,7 and 14 days of first-line anti-TB drug therapy (herein referred to as post-treatment initiation). The mask samples were collected as described in Shaikh et al., 2019, with a slight modification. A detailed description of the sampling approach is provided in the supplementary section. Sputum was collected to confirm the viability of Mtb through culture and identify strain lineage and genotypic drug susceptibility by whole genome sequencing. All patients were followed-up via phone or in-person to monitor treatment adherence and outcome. The outcome, if the patient was cured, was verified through an X-ray by the treating physician. Of the total 50 patients recruited, we present the analysis from 19 patient samples for whom complete data and follow-up profiles were available. Nine patient sample sets were included in the investigation group, and ten patient sample sets in the validation data group. The patient details are described in Supplementary Table S1.

The investigation group included seven DS and two DR patients, while the validation group included 10 DS patients. All patients, including DR patients (whose DR status was unknown at the time of recruitment), were on first-line anti-TB therapy (HRZE) during the first 14 days. All DS patients were declared cured at the end of treatment and hence considered to have received effective treatment, and both DR patients were considered to have received ineffective treatment till 14 days, following which they were shifted to DR treatment based on GeneXpert. In the investigation group, three DS and two DR patient sample sets were individually considered for analysis, while sample sets from four DS patients were pooled at the time-points mentioned above to understand the cumulative effect on transcription. All patient sample sets were analyzed individually for validation.

### Sample processing and RNA sequencing

Membrane peeled from mask samples was collected in RNAzol (Sigma-Aldrich, Missouri, USA) and used for RNA isolation. Sputa were processed by the standard NALC-NaOH method and subjected to BD-BACTEC MGIT for culturing. The total RNA was isolated from RNAzol containing the dissolved gelatin membrane as per the manufacturer's protocol. The RNA obtained was purified using an RNeasy Micro RNA isolation kit (Qiagen, Hilden, Germany). RNA sequencing libraries were prepared with Illumina-compatible NEBNext Ultra II Directional RNA Library Prep Kit (New England BioLabs, Massachusetts, USA) meant for low-input RNA at Genotypic Technology Pvt Ltd., Bangalore, India. The library preparation method has been described in detail in the supplementary section. The libraries were sequenced on the Illumina HiSeq 4000 sequencer (Illumina, San Diego, USA) for 150 bp paired-end chemistry following the manufacturer's procedure.

### Data analysis

Details for the processing of the raw data can be found in the supplementary section. HTSeq^41^ was used to estimate and calculate gene abundance. Absolute counts for genes were identified, which were used in differential expression calculations. DESeq^42^ was used to calculate the differentially expressed genes. Genes were categorized into Up, Down, and Neutrally regulated based on the log2 fold change cut-off of 1.5 value. Supplementary Tables S7 and S8 list genes dysregulated after treatment initiation in the effective treatment (S7) and ineffective treatment groups (S8). We mapped the Mtb transcriptional response to first-line anti-TB therapy — pretreatment and post-treatment initiation using unsupervised principal component analysis (PCA). The PCA was done using the "prcomp" function in R with DEseq normalized counts and visualized using the gplot2 package. To assess the Mtb functions that changed with treatment, we classified genes with altered regulation into functional categories previously described by Mycobrowser and the Cole et al. study^28^. An unadjusted *p*-value < 0.05 was used to describe significantly increased or decreased expression from pretreatment. A modified Fischer exact test^43^ was used to identify the enriched functional category (FDR < 0.05). Enrichment of genes in biological process and KEGG^26^ pathway was performed using Database for Annotation, Visualization and Integrated Discovery (DAVID) web interface v 6.8^27^, with the calculation of fold enrichment, adjusted (FDR) and unadjusted p-value. Genes were considered enriched in a particular process or pathway only if *p*-values and FDR < 0.05. For generating heat maps for the two patient groups, hierarchical clustering using Euclidian distance and average linkage were performed on log2 fold change compared to pretreatment in key genes associated with various functional categories. The heat maps were generated using the Clustvis web tool^44^.

### qRT-PCR validation assay

Real-time RT-qPCR measured the transcripts of 16 selected genes in the RNA isolated from ten validation patient sets. The primers for the validation genes were designed using Primer 3 (see Supplementary Table S9). cDNA was synthesized from 100 ng of total RNA using the Iscript First-Strand Synthesis kit (Biorad, California, USA). After first-strand synthesis, cDNA underwent controlled multiplex amplification using specific flanking primers as previously described^45^. Mtb transcripts were quantified via qRT-PCR using the SYBR Green I Master mix and the CFX96 qPCR system (Biorad). The qRT-PCR was subjected to 40 cycles of amplification, and a melting curve analysis was performed to ensure that the fluorescence levels detected were due to the amplification of a specific product. Normalization of these data was performed by using the *rrs* gene (16 S rRNA) expression levels. The statistical analysis was carried out in GraphPad prism 6 through a two-way ANOVA. *P*-value < 0.05 was considered as significant change.

## Supplementary Information

Supplementary Information 1.

Supplementary Table S6.

Supplementary Table S7.

Supplementary Table S8.

## Data Availability

The processed and aligned Mtb-specific RNA sequences have been deposited in the Sequence Resource Archive (PRJNA717900, with SRA ID: SRR14085853-SRR14058882). All relevant data is provided as supplementary information available on the publisher's website. Any other data is available upon request to the corresponding author.
