# Supplementary material for: Early phase of effective treatment induces distinct transcriptional changes in Mycobacterium tuberculosis expelled by pulmonary tuberculosis patients
Source: Sci Rep. 2021 Sep 8;11:17812. doi: 10.1038/s41598-021-96902-7 (PMC8426492; doi:10.1038/s41598-021-96902-7)
Supplement: Supplementary file 1 — Supplementary Information 1. [file 41598_2021_96902_MOESM1_ESM.docx]

**Early phase of effective treatment induces distinct transcriptional changes in *Mycobacterium tuberculosis* expelled by pulmonary tuberculosis patients**

Ambreen Shaikh^1^, Kalpana Sriraman^1^, Smriti Vaswani^1^, Vikas Oswal^2^, Sudha Rao^3^, Nerges Mistry^1*^

**Supplementary Section**

**Materials and Methods**

Mask Sampling

For mask sampling, the patients wore a modified cup-type N95 mask (3M, Chennai, India) with an attached 37mm diameter gelatin membrane (Sartorius, Gottingen, Germany) on the inner surface of the mask for 10 minutes. The patients undertook specific vocal tasks that included talking/reading, coughing, and tidal breathing for 10 minutes as directed by the sample collector. After completing mask sampling, the membrane was removed from the mask using sterile disposable forceps and transferred to a collection cup containing 2ml of RNAzol (Sigma-Aldrich, Missouri, USA). The collected sample was then transported to the FMR lab at room temperature for further processing.

RNA Sequencing – Library preparation

Total RNA was taken for fragmentation and priming. Fragmented and primed RNA was further subjected to the first-strand synthesis followed by second-strand synthesis. The double-stranded cDNA was purified using HighPrep Beads (Magbio Genomics Inc, Maryland, USA). Purified cDNA was end-repaired, adenylated, and ligated to Illumina multiplex barcode adapters as per NEBNext Ultra II Directional RNA Library Prep protocol followed by second-strand excision using enzyme at 37 ˚C for 15min. Adapter ligated cDNA was purified using HighPrep Beads and was subjected to 16 cycles for Indexing- (98˚C for 30 sec, cycling (98˚C for 10sec, 65˚C for 75sec) and 65˚C for 5min) to enrich the adapter-ligated fragments. The final PCR product (sequencing library) was purified with HighPrep Beads, followed by a library-quality control check. The Illumina-compatible sequencing library was quantified by a Qubit fluorometer (Thermo Fisher Scientific, Massachusetts, USA), and its fragment size distribution was analyzed.

Data Analysis

The data obtained from the sequencing run was de-multiplexed using Bcl2fastq software v2.20, and FastQ files were generated based on the unique dual barcode sequences. The raw reads were processed using FastQC1 for quality assessment and preprocessing, including removing the adapter sequences and low-quality bases (<q30) using Cutadapt2. The preprocessed high-quality data were aligned to reference Homo sapiens and Mycobacterium tuberculosis H37Rv using Bowtie2 with the default parameters to identify the alignment percentage. Reads are classified into aligned reads (which align to the reference genome) and unaligned reads.

Data Normalization in DESeq

As a first step, a pseudo-reference sample was created considering the expression values of a gene across all samples (row-wise geometric mean).

| D | Control_1 | Control_2 | Control_3 | Treated_1 | Treated_2 | Treated_3 | Geo_mean |
| --- | --- | --- | --- | --- | --- | --- | --- |
| gene_2093 | 1 | 4 | 3 | 1 | 2 | 2 | 1.9063685 |
| gene_5153 | 4 | 1 | 1 | 1 | 1 | 2 | 1.4142135 |
| gene_167e | 105 | 88 | 1 | 33 | 48 | 31 | 27.720313 |
| gene_5523 | 34 | 25 | 13 | 26 | 37 | 36 | 26.944697 |
| gene_1425 | 222 | 227 | 197 | 109 | 41 | 77 | 122.72254 |
| gene_2873b | 19 | 9 | 10 | 5 | 8 | 5 | 8.3625299 |
| gene_2106 | 113 | 123 | 124 | 79 | 110 | 100 | 106.96396 |

The next step was to compute the relative expression against the pseudo reference. This is performed for each sample in the dataset. Most genes show no change in expression and are expected to have similar ratios within the sample.

| D | Control_1 | Control_2 | Control_3 | Treated_1 | Treated_2 | Treated_3 |
| --- | --- | --- | --- | --- | --- | --- |
| gene_2093 | 0.524557 | 2.09823 | 1.573672 | 0.524557 | 1.04911506 | 1.04911506 |
| gene_5153 | 2.8284271 | 0.707106 | 0.70710678 | 0.7071067 | 0.7071067 | 1.4142135 |
| gene_167e | 3.78783592 | 3.174567 | 0.0360746 | 1.1904627 | 1.7315821 | 1.1183134 |
| gene_5523 | 1.26184383 | 0.927826 | 0.482469 | 0.964939 | 1.37318299 | 1.33606993 |
| gene_1425 | 1.8089585 | 1.8497 | 1.605247 | 0.888182 | 0.334086 | 0.6274315 |
| gene_2873b | 2.2720397 | 1.07622933 | 1.1958103 | 0.59790518 | 0.9566482 | 0.5979051 |
| gene_2106 | 1.05643052 | 1.14991995 | 1.1592688 | 0.7385664 | 1.0283836 | 0.9348942 |

A normalization factor was then computed as the median value of all ratios for that sample. Differentially expressed genes should not affect the median value.

Normalized counts of genes for a sample were generated by dividing each raw count value in a given sample by that sample's normalization factor. This is performed for all count values (every gene in every sample).

| ID | Control_1 | Control_2 | Control_3 | Treated_1 | Treated_2 | Treated_3 |  |
| --- | --- | --- | --- | --- | --- | --- | --- |
| gene_2093 | 1 | 4 | 3 | 1 | 2 | 2 | Absolute |
| gene_2093 | 0.81393 | 3.7313 | 3.08008 | 1.026588 | 2.08159 | 2.12472 | Normalized |

In the current data set, the control was pretreatment time point.

PCA plot computation

The PCA plot was prepared for individual data sets. First, the z-scores of each value of the variable were calculated to standardize the data. The observations from the mean were subtracted from the mean of the feature to change the origin to the centroid and ensure that PC1 passes through the origin. A covariance matrix was created, and an eigenvector and eigenvalue for the matrix were obtained. We have used the prcomp function of R for generating principle components. ggplot2 package was used for visualization of principal components.

**Supplementary Table S1: Details of patients recruited in the study**

| Investigation Set | | | | | | | | | |  |
| --- | --- | --- | --- | --- | --- | --- | --- | --- | --- | --- |
| Patient data set | **Mtb lineage** | **GeneXpert** | **Treatment profile** | **Treatment Status** | **DR status (WGS)** | **Chest X-Ray** | | **Comorbidities** | **BMI** | |
|  |  |  |  |  |  | Impression | Score |  |  | |
| 1 (M/31) | Delhi CAS | DS-High | Effective | Successfully Completed | Susceptible to HRZE | Moderate | 6 | No | 17 | |
| 2 (M/19) | Delhi CAS | DR-Medium | Ineffective | Shifted to correct Rx | Resistant to HRZE, Fq, Streptomycin | Moderate | 10 | No | 18 | |
| 3 (M/20) | European American | DS-Medium | Effective | Successfully Completed | Susceptible to HRZE | Mild | 4 | No | 21 | |
| 4 (M/21) | European American | DS-Medium | Effective | Successfully Completed | Susceptible to HRZE | Moderate | 11 | No | 18 | |
| 5 (F/28) | Beijing | DR-Medium | Ineffective | Shifted to correct Rx | Resistant to HRZE, Fq, Streptomycin | Mild | 6 | Yes (Diabetes) | 24 | |
| Pooled Set (4F/Median age 28) | Beijing + Delhi CAS + European American | Combination of DS samples | Effective | Successfully Completed | Susceptible to HRZE | Moderate | 8.5 | No | 20 | |
|  |  |  |  |  |  |  |  |  |  | |
| Validation Set | | | | | | | | | |  |
| 1 (F/21) | Delhi-CAS | DS-Low | Effective | Successfully Completed | Susceptible to HRZE | Moderate | 7 | No | 19 | |
| 2 (M/45) | Delhi-CAS | DS-High | Effective | Successfully Completed | Susceptible to HRZE | Extensive | 9 | Yes (Diabetes) | 22 | |
| 3 (M/23) | Delhi-CAS | DS-High | Effective | Successfully Completed | Susceptible to HRZE | Moderate | 10 | NT | 19 | |
| 4 (M/18) | Delhi-CAS | DS-High | Effective | Successfully Completed | Susceptible to HRZE | Mild | 6 | NT | 19 | |
| 5 (M/46) | East African Indian Ocean | DS-High | Effective | Successfully Completed | Susceptible to HRZE, Resistant to Fq | Moderate | 9 | No | 21 | |
| 6 (F/18) | Delhi-CAS | DS-High | Effective | Successfully Completed | Susceptible to HRZE | Moderate | 12 | NT | 13 | |
| 7 (M/19) | European American | DS-Medium | Effective | Successfully Completed | Susceptible to HRZE, Resistant to Streptomycin | Moderate | 9 | NT | 18 | |
| 8 (F/25) | - | DS-Low | Effective | Successfully Completed | - | Moderate | 8 | NT | 24 | |
| 9 (M/21) | European American | DS-Medium | Effective | Successfully Completed | Susceptible to HRZE | Moderate | 7 | NT | 18 | |
| 10 (M/19) | Delhi-CAS | DS-Medium | Effective | Successfully Completed | Susceptible to HRZE | Moderate | 9 | NT | 14 | |

The values in the bracket in column 1 represent gender and age, HRZE- Isoniazid, Rifampicin, Pyrazinamide, Ethambutol, Fq-Fluoroquinolones, NT- Not tested for HIV or Diabetes, DS- Drug Susceptible, DR-Drug Resistant, Rx- treatment

**Supplementary Figure S1**


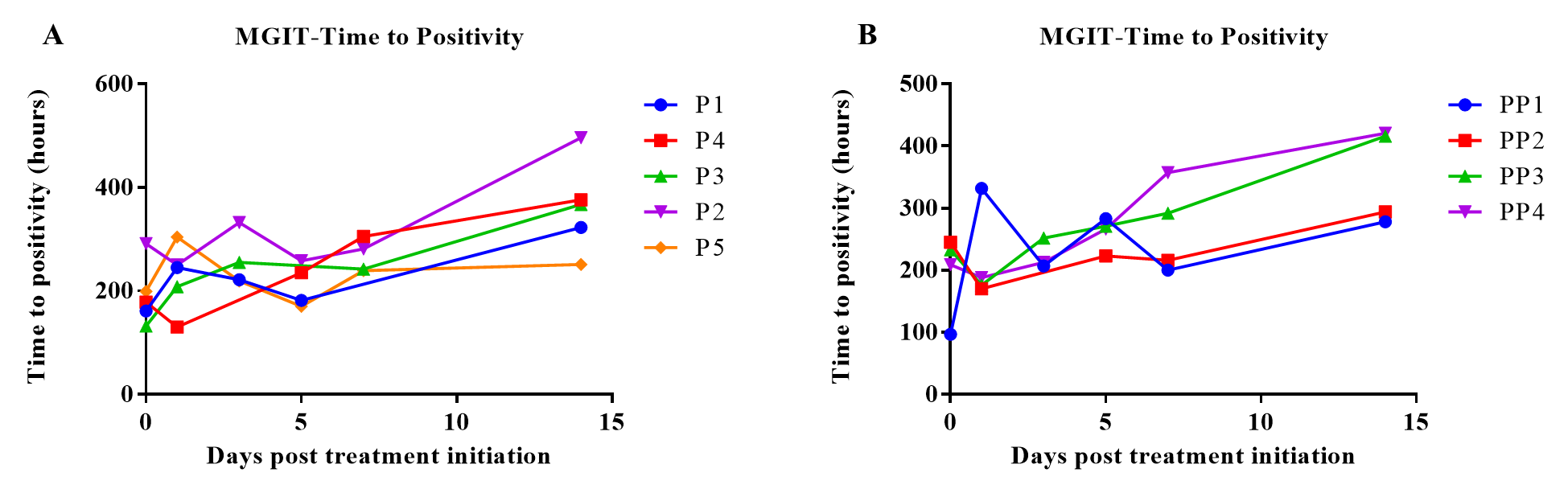


**Figure S1: Time to Positivity (TTP) in MGIT with days of treatment** - The graphs depict the time to positivity (TTP) in MGIT on each day post-treatment initiation in (A) Investigation set – patient set 1-5 and (B) Pooled patient set. Each line in the graphs represents the TTP of a single patient set. The time to positivity increases with treatment days. TTP was tracked from Mtb isolated from patients' sputa in the investigative set collected pretreatment and completing 1,3,5,7 and 14 days of treatment. P1-P5- Patient Set, PP1-PP4 – Pooled Patient Set

**Supplementary Table S2 - Summary of RNA sequencing analysis of patient sets (n=9)**

|  | Treatment Time points | | | | | |
| --- | --- | --- | --- | --- | --- | --- |
|  | **Pre-Rx** | **Rx-1D** | **Rx-3D** | **Rx-5D** | **Rx-7D** | **Rx-14D** |
| Processed Reads | 3.96E+07 (2.76E+07 - 4.47E+07) | 4.35E+07 (2.5.9E+07 - 4.84E+07) | 3.63E+07 (2.96E+07 - 4.46E+07) | 3.64E+07 (2.99E+07 - 4.64E+07) | 2.71E+07 (2.76E+07 - 4.52E+07) | 3.35E+07  (2.61E+07 - 4.41E+07) |
| Mtb percentage | 1.51 (0.16-22.88) | 2.7 (0.78 -5.72) | 0.66 (0.31-2.27) | 1.44 (0.43 - 2.48) | 0.55 (0.35 - 1.03) | 0.44 (0.34- 1) |
| Mtb Specific reads | 590217  (65229-4731000) | 935018  (350276 - 1765000) | 240696  (93235 - 2422000) | 541793  (201794 -917478) | 174478 (124540 - 411382) | 195536  (94393 -335060) |
| Genes covered | 1988 (130 - 3925) | 833 (229 - 2719) | 303 (93 -1049) | 273 (201 - 2041) | 425 (203 - 1089) | 410 (127 - 1366) |
|  |  |  |  |  |  |  |

Pre Rx- Pretreatment, Rx-1d – Rx-14d – Days post-treatment initiation, Mtb –*Mycobacterium tuberculosis*

**Supplementary Figure S2**


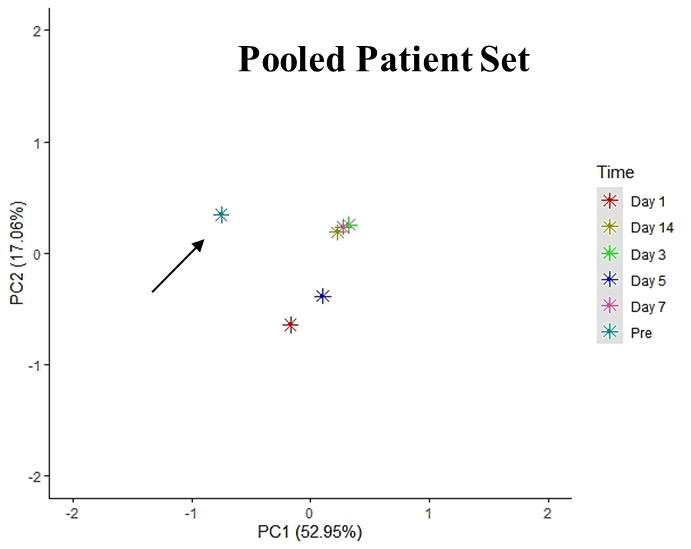


**Figure S2:** Principal Component Analysis (PCA) – The PCA plot with the first and second principal components for the pooled patient set is shown. Each point in the plots represents a transcriptional signature profiled on a particular time point pre-and post-treatment initiation. The arrow marks the pretreatment point in the plot. The sample was pooled by combining the RNA from 4 patient data set at each profiled time point.

**Supplementary Table S3- Summary of transcripts detected in the two treatment groups**

| Treatment Groups | Treatment Time point | Total | Dysregulation | Upregulation | Down-Regulation | Neutral Expression |
| --- | --- | --- | --- | --- | --- | --- |
| Effective Treatment | Pre-Rx | 3980 |  |  |  |  |
|  | Rx-1d | 3959 | 287 | 166 | 121 | 3672 |
|  | Rx-3d | 1458 | 591 | 318 | 273 | 867 |
|  | Rx-5d | 3504 | 1439 | 710 | 729 | 2065 |
|  | Rx-7d | 491 | 266 | 136 | 130 | 225 |
|  | Rx-14d | 265 | 157 | 81 | 76 | 108 |
|  |  |  |  |  |  |  |
| Ineffective Treatment | Pre-Rx | 404 |  |  |  |  |
|  | Rx-1d | 350 | 150 | 68 | 82 | 200 |
|  | Rx-3d | 300 | 120 | 55 | 65 | 180 |
|  | Rx-5d | 72 | 30 | 11 | 19 | 42 |
|  | Rx-7d | 87 | 28 | 18 | 10 | 59 |
|  | Rx-14d | 53 | 27 | 19 | 8 | 26 |

Rx- Treatment, Rx-1D to Rx-14D – days post-treatment initiation

**Supplementary Table S4 - Classification of genes as per functional categories describe by Mycobrowser**

| Functional Categories |  | Rx-1D | | | | Rx-3D | | | | Rx-5D | | | | Rx-7D | | | | Rx-14D | | | |
| --- | --- | --- | --- | --- | --- | --- | --- | --- | --- | --- | --- | --- | --- | --- | --- | --- | --- | --- | --- | --- | --- |
| Effective treatment | | | | | | | | | | | | | | | | | | | | | |
|  | Total | Up | % | Down | % | Up | % | Down | % | Up | % | Down | % | Up | % | Down | % | Up | % | Down | % |
| Cell wall and Cell wall process | 688 | 23 | 3.34 | 17 | 2.47 | 76 | 11.05 | 49 | 7.12 | 230 | 33.43 | 20 | 2.91 | 27 | 3.92 | 25 | 3.63 | 11 | 1.60 | 11 | 1.60 |
| Conserved hypotheticals | 1021 | 43 | 4.21 | 33 | 3.23 | 85 | 8.33 | 41 | 4.02 | 211 | 20.67 | 189 | 18.51 | 33 | 3.23 | 15 | 1.47 | 20 | 1.96 | 10 | 0.98 |
| Information pathways | 227 | 4 | 1.76 | 11 | 4.85 | 7 | 3.08 | 34 | 14.98 | 19 | 8.37 | 59 | 25.99 | 5 | 2.20 | 13 | 5.73 | 6 | 2.64 | 5 | 2.20 |
| Insertion Sequences and Phages | 74 | 12 | 16.22 | 4 | 5.41 | 6 | 8.11 | 1 | 1.35 | 22 | 29.73 | 5 | 6.76 | 5 | 6.76 | 0 | 0.00 | 1 | 1.35 | 0 | 0.00 |
| Intermediary metabolism and Respiration | 836 | 24 | 2.87 | 15 | 1.79 | 58 | 6.94 | 60 | 7.18 | 122 | 14.59 | 151 | 18.06 | 30 | 3.59 | 27 | 3.23 | 21 | 2.51 | 9 | 1.08 |
| Lipid Metabolism | 224 | 1 | 0.45 | 9 | 4.02 | 16 | 7.14 | 32 | 14.29 | 43 | 19.20 | 47 | 20.98 | 6 | 2.68 | 19 | 8.48 | 6 | 2.68 | 12 | 5.36 |
| Not Classified | 398 | 36 | 9.05 | 12 | 3.02 | 21 | 5.28 | 30 | 7.54 | 66 | 16.58 | 69 | 17.34 | 5 | 1.26 | 16 | 4.02 | 8 | 2.01 | 16 | 4.02 |
| PE/PPE | 157 | 11 | 7.01 | 1 | 0.64 | 13 | 8.28 | 8 | 5.10 | 50 | 31.85 | 22 | 14.01 | 11 | 7.01 | 5 | 3.18 | 5 | 3.18 | 3 | 1.91 |
| Regulatory Proteins | 178 | 10 | 5.62 | 4 | 2.25 | 23 | 12.92 | 7 | 3.93 | 40 | 22.47 | 34 | 19.10 | 9 | 5.06 | 3 | 1.69 | 2 | 1.12 | 2 | 1.12 |
| Virulence Detoxification Adaptation | 99 | 5 | 5.05 | 9 | 9.09 | 11 | 11.11 | 11 | 11.11 | 20 | 20.20 | 18 | 18.18 | 5 | 5.05 | 7 | 7.07 | 1 | 1.01 | 7 | 7.07 |
| Ineffective treatment | | | | | | | | | | | | | | | | | | | | | |
| Cell wall and Cell wall process | 688 | 11 | 1.60 | 9 | 1.31 | 4 | 0.58 | 10 | 1.45 | 2 | 0.29 | 3 | 0.44 | 3 | 0.44 | 1 | 0.15 | 4 | 0.58 | 0 | 0.00 |
| Conserved hypotheticals | 1021 | 10 | 0.98 | 16 | 1.57 | 13 | 1.27 | 7 | 0.69 | 2 | 0.20 | 3 | 0.29 | 4 | 0.39 | 0 | 0.00 | 2 | 0.20 | 0 | 0.00 |
| Information pathways | 227 | 8 | 3.52 | 6 | 2.64 | 4 | 1.76 | 11 | 4.85 | 2 | 0.88 | 2 | 0.88 | 0 | 0.00 |  | 0.00 | 4 | 1.76 | 1 | 0.44 |
| Insertion Sequences and Phages | 74 | 1 | 1.35 | 1 | 1.35 | 0 | 0.00 | 1 | 1.35 | 0 | 0.00 |  | 0.00 | 0 | 0.00 |  | 0.00 | 0 | 0.00 |  | 0.00 |
| Intermediary metabolism and Respiration | 836 | 12 | 1.44 | 23 | 2.75 | 10 | 1.20 | 18 | 2.15 | 3 | 0.36 | 0 | 0.00 | 7 | 0.84 | 4 | 0.48 | 5 | 0.60 | 1 | 0.12 |
| Lipid Metabolism | 224 | 9 | 4.02 | 10 | 4.46 | 7 | 3.13 | 7 | 3.13 | 1 | 0.45 | 6 | 2.68 | 2 | 0.89 | 0 | 0.00 | 1 | 0.45 | 1 | 0.45 |
| Not classified | 398 | 8 | 2.01 | 6 | 1.51 | 8 | 2.01 | 3 | 0.75 | 1 | 0.25 | 5 | 1.25 | 2 | 0.50 | 2 | 0.50 | 2 | 0.50 | 3 | 0.75 |
| PE/PPE | 157 | 5 | 3.18 | 5 | 3.18 | 5 | 3.18 | 6 | 3.82 | 0 | 0.00 |  | 0.00 | 2 | 1.27 | 1 | 0.64 | 0 | 0.00 | 1 | 0.64 |
| Regulatory Proteins | 178 | 3 | 1.69 | 4 | 2.25 | 1 | 0.56 | 1 | 0.56 | 0 | 0.00 |  | 0.00 | 0 | 0.00 |  | 0.00 | 0 | 0.00 | 1 | 0.56 |
| Virulence Detoxification Adaptation | 99 | 2 | 2.02 | 2 | 2.02 | 3 | 3.03 | 1 | 1.01 | 0 | 0.00 | 0 | 0.00 | 0 | 0.00 | 0 | 0.00 | 1 | 1.01 | 0 | 0.00 |

Total – total numbers of genes detected in the category; up – number of genes upregulated; down- number of genes down-regulated. %- the percentage of genes up and down-regulated in each category depending on the day of treatment in each treatment group; Rx- Treatment, Rx-1D to Rx-14D – days post-treatment initiation. All highlighted squares represent significant up or downregulation of genes in the functional category based on modified Fischer exact test, p < 0.05 – significant.

**Supplementary Table S5 – Enrichment Analysis of Biological Processes and KEGG pathways**

| **Effective Treatment - Downregulation** | | | | | |
| --- | --- | --- | --- | --- | --- |
| **Days post Rx initiation** | **GO term** | **Count** | **p-value** | **Fold enrichment** | **FDR** |
| Rx-1D | Growth | 34 | 5.60E-04 | 1.70 | 3.40E-02 |
|  | Response to Hypoxia | 11 | 2.90E-06 | 6.60 | 3.50E-04 |
|  | Pathogenesis | 13 | 1.10E-03 | 2.90 | 4.40E-02 |
|  | Cellular Response to Starvation | 3 | 2.70E-02 | 11.00 | 8.20E-01 |
|  | Tricarboxylic Acid Cycle | 4 | 5.00E-02 | 4.70 | 9.40E-01 |
|  |  |  |  |  |  |
| Rx-3D | Growth | 93 | 7.60E-09 | 1.7 | 1.80E-06 |
|  | Pathogenesis | 34 | 4.40E-08 | 2.7 | 5.20E-06 |
|  | Protein Secretion by the Type VII Secretion System | 7 | 1.00E-04 | 7.4 | 7.10E-03 |
|  | Response to Heat | 8 | 1.20E-04 | 6 | 7.10E-03 |
|  | Response to Hypoxia | 12 | 4.80E-03 | 2.6 | 2.30E-01 |
|  | Fatty Acid Biosynthetic Process | 11 | 7.70E-03 | 2.6 | 3.00E-01 |
|  | Transport | 12 | 1.60E-02 | 2.2 | 5.30E-01 |
|  | DNA Topological Change | 3 | 2.50E-02 | 10.5 | 7.40E-01 |
|  | Response to Oxidative Stress | 6 | 3.50E-02 | 3.2 | 9.10E-01 |
|  |  |  |  |  |  |
| Rx-5D | Translation | 32 | 2.20E-06 | 2.30E+00 | 6.40E-04 |
|  | Growth | 161 | 3.10E-06 | 1.3 | 6.40E-04 |
|  | Response to Hypoxia | 25 | 6.90E-06 | 2.5 | 9.60E-04 |
|  | Pathogenesis | 43 | 1.00E-03 | 1.6 | 1.00E-01 |
|  | Mycolate Cell Wall Layer Assembly | 8 | 2.30E-03 | 3.5 | 1.90E-01 |
|  | Protein Secretion by the Type VII Secretion System | 7 | 7.30E-03 | 3.4 | 5.00E-01 |
|  | Response to Iron Ion | 6 | 1.20E-02 | 3.6 | 6.90E-01 |
|  | Cellular Response to Phosphate Starvation | 4 | 2.90E-02 | 4.8 | 1.00E+00 |
|  |  |  |  |  |  |
| Rx-7D | Growth | 44 | 6.50E-06 | 1.8 | 1.00E-03 |
|  | Response to Hypoxia | 9 | 6.00E-04 | 4.5 | 4.80E-02 |
|  | Response to Heat | 5 | 1.80E-03 | 8.7 | 9.70E-02 |
|  | Response to Antibiotic | 7 | 2.70E-02 | 3 | 8.60E-01 |
|  | Actinobacterium-Type Cell Wall Biogenesis | 4 | 2.90E-02 | 5.8 | 8.60E-01 |
|  | Pathogenesis | 11 | 3.50E-02 | 2.1 | 8.60E-01 |
|  | Growth of Symbiont in Host | 5 | 3.80E-02 | 3.8 | 8.60E-01 |
|  |  |  |  |  |  |
| Rx-14D | Protein Secretion by The type VII Secretion System | 5 | 6.30E-05 | 19.9 | 5.70E-03 |
|  | Growth | 25 | 3.60E-03 | 1.7 | 1.30E-01 |
|  | Fatty Acid Biosynthetic Process | 6 | 4.40E-03 | 5.3 | 1.30E-01 |
|  | DIM/DIP Cell Wall Layer Assembly | 4 | 1.00E-02 | 8.4 | 2.40E-01 |
|  | Response to Hypoxia | 5 | 3.10E-02 | 4.1 | 5.60E-01 |
|  | Pathogenesis | 8 | 3.90E-02 | 2.4 | 6.00E-01 |
|  |  |  |  |  |  |
|  | **KEGG Pathway** |  |  |  |  |
| Rx-1D | Citrate Cycle (TCA) | 5 | 1.70E-02 | 4.70 | 6.80E-01 |
|  | Ribosome | 6 | 3.20E-02 | 3.20 | 6.80E-01 |
|  |  |  |  |  |  |
| Rx-3D | RNA Degradation | 6 | 2.00E-03 | 5.8 | 6.50E-02 |
|  | Tuberculosis | 6 | 2.00E-03 | 5.8 | 6.50E-02 |
|  | Fatty Acid Biosynthesis | 6 | 6.10E-03 | 4.6 | 1.30E-01 |
|  | Carbon Metabolism | 18 | 8.20E-03 | 1.9 | 1.30E-01 |
|  | Microbial Metabolism in Diverse Environments | 27 | 1.60E-02 | 1.5 | 1.70E-01 |
|  | Pyruvate Metabolism | 10 | 1.70E-02 | 2.4 | 1.70E-01 |
|  | Purine Metabolism | 12 | 2.00E-02 | 2.1 | 1.70E-01 |
|  | Citrate Cycle (TCA Cycle) | 8 | 2.10E-02 | 2.7 | 1.70E-01 |
|  | Fatty Acid Metabolism | 10 | 2.70E-02 | 2.2 | 2.00E-01 |
|  | Nitrogen Metabolism | 6 | 3.30E-02 | 3.2 | 2.10E-01 |
|  | RNA Polymerase | 3 | 3.90E-02 | 8.7 | 2.10E-01 |
|  | Glycolysis / Gluconeogenesis | 8 | 4.20E-02 | 2.4 | 2.10E-01 |
|  | Pyrimidine Metabolism | 8 | 4.20E-02 | 2.4 | 2.10E-01 |
|  |  |  |  |  |  |
| Rx-5D | Ribosome | 33 | 5.20E-09 | 2.7 | 4.80E-07 |
|  | Tuberculosis | 8 | 3.70E-03 | 3.3 | 1.70E-01 |
|  | Bacterial Secretion System | 7 | 4.30E-02 | 2.5 | 1.00E+00 |
|  |  |  |  |  |  |
| Rx-7D | No Enrichment |  |  |  |  |
|  |  |  |  |  |  |
| Rx-14D | Fatty Acid Biosynthesis | 4 | 2.80E-03 | 12.5 | 1.00E-01 |
|  |  |  |  |  |  |
| **Ineffective Treatment - Downregulation** | | | | | |
| **Days post Rx initiation** | **GO term** | **Count** | **p-value** | **Fold enrichment** | **FDR** |
| Rx-1D | Growth | 26 | 8.10E-03 | 1.60 | 7.80E-01 |
|  | Negative Regulation of Transcription, DNA-Template | 4 | 3.20E-02 | 5.50 | 1.00E+00 |
|  |  |  |  |  |  |
| Rx-3D | Cell Division | 4 | 2.20E-02 | 6.40 | 1.00E+00 |
|  |  |  |  |  |  |
| Rx-5D | Fatty Acid Biosynthetic Process | 3 | 2.00E-02 | 12.40 | 4.90E-01 |
|  | Growth | 7 | 4.30E-02 | 2.20 | 5.40E-01 |
|  |  |  |  |  |  |
| Rx-7D | Growth | 6 | 3.40E-02 | 2.50 | 8.40E-01 |
|  |  |  |  |  |  |
| Rx-14D | No Enrichment |  |  |  |  |
|  |  |  |  |  |  |
|  | **KEGG Pathway** |  |  |  |  |
| Rx-1D | Alanine, aspartate and glutamate metabolism | 4 | 3.40E-02 | 5.30 | 1.00E+00 |
|  |  |  |  |  |  |
| Rx-3D | No enriched pathways |  |  |  |  |
|  |  |  |  |  |  |
| Rx-5D | Ribosome | 3 | 4.40E-02 | 7.20 | 5.30E-02 |
|  |  |  |  |  |  |
| Rx-7D | No enriched pathways |  |  |  |  |
|  |  |  |  |  |  |
| Rx-14D | No enriched pathways |  |  |  |  |
|  |  |  |  |  |  |
| **Effective Treatment - Upregulation** | | | | | |
| **Days post Rx initiation** | **GO term** | **Count** | **p-value** | **Fold enrichment** | **FDR** |
| Rx-1D | Negative Regulation of Growth | 6 | 7.10E-03 | 4.8 | 5.80E-01 |
|  | Cobalamin Biosynthetic Process | 4 | 1.90E-02 | 6.7 | 5.90E-01 |
|  | Transcription, DNA-Template | 12 | 2.20E-02 | 2.1 | 5.90E-01 |
|  | Regulation of Transcription, DNA-Template | 11 | 3.40E-02 | 2.1 | 6.90E-01 |
|  |  |  |  |  |  |
| Rx-3D | Transcription, DNA-Template | 22 | 1.10E-02 | 1.7 | 1.00E+00 |
|  | Regulation of Transcription, DNA -Template | 19 | 4.20E-02 | 1.6 | 1.00E+00 |
|  |  |  |  |  |  |
| Rx-5D | Transcription, DNA-Template | 41 | 2.50E-03 | 1.6 | 6.30E-01 |
|  | Methylation | 14 | 7.20E-03 | 2.2 | 9.10E-01 |
|  | Transmembrane Transport | 12 | 3.70E-02 | 1.9 | 1.00E+00 |
|  |  |  |  |  |  |
| Rx-7D | Biosynthetic Process | 4 | 3.20E-02 | 5.6 | 1.00E+00 |
|  |  |  |  |  |  |
| Rx-14D | No Enrichment |  |  |  |  |
|  |  |  |  |  |  |
|  | **KEGG Pathway** |  |  |  |  |
| Rx-1D | Ribosome | 5 | 3.60E-02 | 3.7 | 8.40E-01 |
|  |  |  |  |  |  |
| Rx3D | No Enriched Pathways |  |  |  |  |
|  |  |  |  |  |  |
| Rx-5D | ABC Transporters | 24 | 4.40E-05 | 2.4 | 4.00E-03 |
|  | Glycerophospholipid Metabolism | 8 | 1.10E-02 | 3 | 4.90E-01 |
|  | Porphyrin And Chlorophyll Metabolism | 9 | 4.30E-02 | 2.2 | 1.00E+00 |
|  |  |  |  |  |  |
| Rx-7D | No Enriched Pathways |  |  |  |  |
|  |  |  |  |  |  |
| Rx-14D | No Enriched Pathways |  |  |  |  |
|  |  |  |  |  |  |
| **Ineffective Treatment - Upregulation** | | | | | |
|  |  |  |  |  |  |
| **Days post Rx initiation** | **GO term** | **Count** | **p-value** | **Fold enrichment** | **FDR** |
| Rx-1D | Response to Antibiotics | 6 | 5.50E-03 | 5.01 | 5.60E-01 |
|  |  |  |  |  |  |
| Rx-3D | Growth | 19 | 1.80E-03 | 2.00 | 1.30E-01 |
|  |  |  |  |  |  |
| Rx-5D | No Enrichment |  |  |  |  |
|  |  |  |  |  |  |
| Rx-7D | No Enrichment |  |  |  |  |
|  |  |  |  |  |  |
| Rx-14D | No Enrichment |  |  |  |  |
|  |  |  |  |  |  |
|  | **KEGG Pathway** |  |  |  |  |
| Rx-1D | No Enriched Pathways |  |  |  |  |
|  |  |  |  |  |  |
| Rx-3D | Metabolic Pathways | 12 | 2.00E-03 | 1.80 | 8.30E-02 |
|  | Biosynthesis of Secondary Metabolites | 8 | 1.90E-02 | 2.30 | 3.60E-01 |
|  | Biosynthesis of Amino Acids | 5 | 2.60E-02 | 3.70 | 3.60E-01 |
|  |  |  |  |  |  |
| Rx-5D | No Enriched Pathways |  |  |  |  |
|  |  |  |  |  |  |
| Rx-7D | Biosynthesis of Antibiotics | 5 | 2.40E-03 | 4.50 | 4.20E-02 |
|  |  |  |  |  |  |
| Rx-14D | No Enriched Pathways |  |  |  |  |

Rx- Treatment, Rx-1D to Rx-14D – days post-treatment initiation, p-Value < 0.05 considered significant, FDR – False Discovery Rate

Supplementary Tables S6, S7, and S8 are available as separate excel sheets.

**Supplementary Table S9 – Primer Sequences used for validating the expression of key genes**

| Primer Name | Primer sequence |
| --- | --- |
| *moxR3-F* | ggttggaactgcgctacctc |
| *moxR3-R* | gacctgctcgaccgattcac |
| *groEL2-F* | ctcgagctcaccgagggtat |
| *groEL2-R* | cagcaggatgtaggggtcct |
| *mts2823-F* | cacccacgcggagtcatag |
| *mts2823-R* | ctgtagccccacccaaagg |
| *pks13-F* | gatgggcaagagcctgtacc |
| *pks13-R* | gatggtgacctgggtggtct |
| *eccC5-F* | caccgacctcgaagaagacc |
| *eccC5-R* | ggctcgcaccgagttgtact |
| *katG-F* | ctccgagtcactgaccaacg |
| *katG-R* | gagttggacccgaagaccag |
| *espB-F* | ctaggcgggaacatccgact |
| *espB-R* | caccttctccgacctctgct |
| *fadD26-F* | accactaccccgacgacatc |
| *fadD26-R* | ggtgacctcacgcttcacc |
| *bfrB-F* | gctcgtgcaacacctgctc |
| *bfrB-R* | gtcggtgactgtgcgttcct |
| *arsB2-F* | cgcgactctggtgttagtgc |
| *arsB2-R* | cacagtcccagtcgggtgta |

**Supplementary Table S10 – Summary of transcripts detected in drug-induced specific gene clusters**

| Drug treatment | Days post-treatment | Total Number of transcripts | Transcripts detected | Upregulated | Downregulated |
| --- | --- | --- | --- | --- | --- |
| Rifampicin treatment | Rx-1D | 26 | 25 | 1 | 3 |
|  | Rx-3D | 26 | 12 | 0 | 6 |
|  | Rx-5D | 26 | 21 | 1 | 6 |
|  | Rx-7D | 26 | 5 | 2 | 3 |
|  | Rx-14D | 26 | 4 | 0 | 2 |
| Isoniazid Treatment | Rx-1D | 48 | 48 | 0 | 4 |
|  | Rx-3D | 48 | 29 | 2 | 17 |
|  | Rx-5D | 48 | 47 | 2 | 33 |
|  | Rx-7D | 48 | 13 | 0 | 9 |
|  | Rx-14D | 48 | 8 | 0 | 6 |
| Ethambutol treatment | Rx-1D | 12 | 12 | 0 | 1 |
|  | Rx-3D | 12 | 7 | 0 | 7 |
|  | Rx-5D | 12 | 12 | 0 | 7 |
|  | Rx-7D | 12 | 4 | 0 | 3 |
|  | Rx-14D | 12 | 4 | 0 | 4 |

Rx- Treatment, Rx-1D to Rx-14D – days post-treatment initiation

**
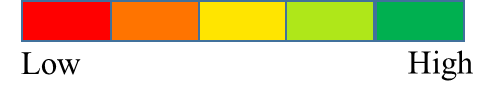
Supplementary Figure S3**

|  |  | Rx-1D | Rx-3D | Rx-5D | Rx-7D | Rx-14D |
| --- | --- | --- | --- | --- | --- | --- |
| Isoniazid treatment | *bfrB* |  |  |  |  |  |
|  | *acpM* |  |  |  |  |  |
|  | *accD6* |  |  |  |  |  |
|  | *iniB* |  |  |  |  |  |
|  | *gyrB* |  |  |  |  |  |
|  | *FAS* |  |  |  |  |  |
|  | *pks13* |  |  |  |  |  |
|  | *pks16* |  |  |  |  |  |
|  | *efpA* |  |  |  |  |  |
|  | *Rv1566c* |  |  |  |  |  |
|  | *sigD* |  |  |  |  |  |
|  | *fadD32* |  |  |  |  |  |
|  | *fabD* |  |  |  |  |  |
|  | *Rv0312* |  |  |  |  |  |
|  | *Rv2166c* |  |  |  |  |  |
|  | *accD4* |  |  |  |  |  |
|  | *Rv2721c* |  |  |  |  |  |
|  | *wag31* |  |  |  |  |  |
|  | *moeY* |  |  |  |  |  |
|  | *fbpC* |  |  |  |  |  |
|  | *Rv3064c* |  |  |  |  |  |
| Rifampicin Treatment | *nrdH* |  |  |  |  |  |
|  | *groEL2* |  |  |  |  |  |
|  | *dnaK* |  |  |  |  |  |
|  | *echA21* |  |  |  |  |  |
|  | *dnaJ1* |  |  |  |  |  |
|  | *Rv0250c* |  |  |  |  |  |
|  | *groES* |  |  |  |  |  |
| Ethambutol treatment | *fabD* |  |  |  |  |  |
|  | *desA2* |  |  |  |  |  |
|  | *desA1* |  |  |  |  |  |
|  | *ppsE* |  |  |  |  |  |
|  | *kasA* |  |  |  |  |  |

**Figure S3** – Heat map - the expression levels of genes belonging to specific drug-induced gene clusters were mapped post days 1-14 of treatment in aerosolized Mtb. The column on the extreme left specifies the drug treatment, and the heat map was generated using log2 fold change values. ND-not detected, Rx- Treatment, Rx-1D to Rx-14D – days post-treatment initiation, blank squares denote transcripts not detected

**Supplementary Figure S4**

**
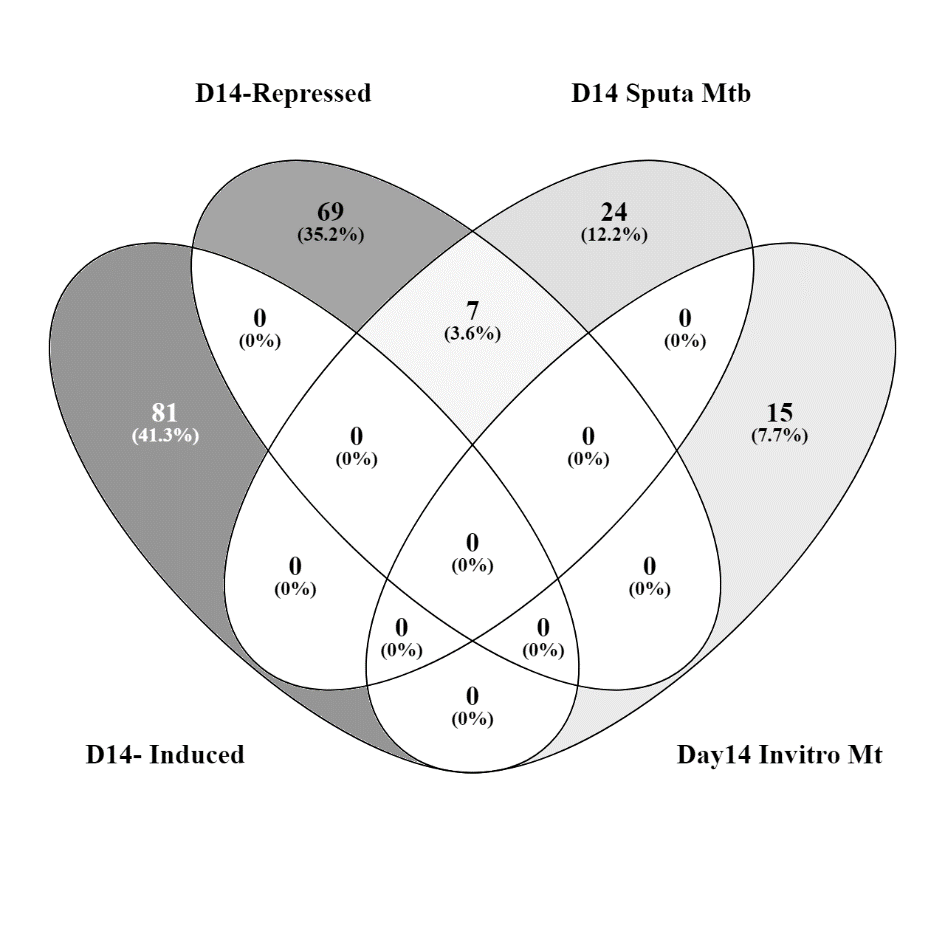
**

**Figure S4** – Venn diagram compares transcriptional signature of aerosolized Mtb, sputa derived Mtb on day 14 after exposure to antimycobacterial drugs *in vivo* and *in vitro.* The diagram shows the overlap of the number of genes in these transcriptional signatures. D14-Repressed- genes repressed in aerosolized drug-sensitive Mtb on day 14 after exposure to standard first-line treatment *in vivo*. D14-Induced- genes induced in aerosolized drug-sensitive Mtb on day 14 after exposure to standard first-line treatment *in vivo*. D14 Sputa Mtb - genes repressed in sputa derived Mtb on day 14 after exposure to standard treatment *in vivo^1^*. Day14 in vitro Mtb - genes repressed sputa derived Mtb with 14 days' exposure to rifampicin, isoniazid, ciprofloxacin, and pyrazinamide *in vitro^2^*.

**Supplementary Figure S5**

Figure S5 – The graph shows the expression pattern of Mtb genes commonly repressed in aerosolized and sputum Mtb on day 14 when mapped in aerosolized Mtb on different days of treatment. Log2 expression ratios are plotted for 1,3,5,7 and 14 days after the start of standard first-line treatment; the y-axis details fold change relative to day 0.

**References**

1 Honeyborne, I. *et al.* Profiling persistent tubercule bacilli from patient sputa during therapy predicts early drug efficacy. *BMC medicine* **14**, 1-13 (2016).

2 Keren, I., Minami, S., Rubin, E. & Lewis, K. Characterization and transcriptome analysis of Mycobacterium tuberculosis persisters. *MBio* **2** (2011).
